# Loop-Mediated Isothermal Amplification (LAMP) Assay for Rapid and Accurate Confirmatory Diagnosis of HTLV-1/2 Infection

**DOI:** 10.3390/v12090981

**Published:** 2020-09-04

**Authors:** Yago Gomes, Adele Caterino-de-Araujo, Karoline Campos, Maria Gisele Gonçalves, Ana Claudia Leite, Marco Antonio Lima, Abelardo Araújo, Marcus Tulius Silva, Otávio Espíndola

**Affiliations:** 1 Laboratory for Clinical Research in Neuroinfections, Evandro Chagas National Institute of Infectious Diseases (INI), Oswaldo Cruz Foundation (FIOCRUZ), Rio de Janeiro 21040-900, Brazil; ana.leite@ini.fiocruz.br (A.C.L.); marco.lima@ini.fiocruz.br (M.A.L.); abelardo.araujo@gmail.com (A.A.); 2 Laboratory of HTLV Research, Immunology Center, Adolfo Lutz Institute, São Paulo 01246-000, Brazil; caterino@alumni.usp.br (A.C.-d.-A.); karol_rodriguescamp@yahoo.com.br (K.C.); giselegoncalvesial@yahoo.com.br (M.G.G.)

**Keywords:** HTLV-1, HTLV-2, LAMP, confirmatory diagnosis

## Abstract

Laboratory diagnosis of human T-lymphotropic viruses (HTLV) 1 and 2 infection is performed by serological screening and further confirmation with serological or molecular assays. Thus, we developed a loop-mediated isothermal nucleic acid amplification (LAMP) assay for the detection of HTLV-1/2 in blood samples. The sensitivity and accuracy of HTLV-1/2 LAMP were defined with DNA samples from individuals infected with HTLV-1 (*n* = 125), HTLV-2 (*n* = 19), and coinfected with HIV (*n* = 82), and compared with real-time polymerase chain reaction (qPCR) and PCR-restriction fragment length polymorphism (RFLP). The overall accuracy of HTLV-1/2 LAMP (95% CI 74.8–85.5%) was slightly superior to qPCR (95% CI 69.5–81.1%) and similar to PCR-RFLP (95% CI 79.5–89.3%). The sensitivity of LAMP was greater for HTLV-1 (95% CI 83.2–93.4%) than for HTLV-2 (95% CI 43.2–70.8%). This was also observed in qPCR and PCR-RFLP, which was associated with the commonly lower HTLV-2 proviral load. All molecular assays tested showed better results with samples from HTLV-1/2 mono-infected individuals compared with HIV-coinfected patients, who present lower CD4 T-cell counts. In conclusion, HTLV-1/2 LAMP had similar to superior performance than PCR-based assays, and therefore may represent an attractive alternative for HTLV-1/2 diagnosis due to reduced working time and costs, and the simple infrastructure needed.

## 1. Introduction

Human T-lymphotropic virus (HTLV) type 1 and 2 are retroviruses transmitted among humans through the parenteral route, unprotected sexual intercourse, and from mother-to-child, particularly by breastfeeding [1]. The global distribution of these viruses is heterogeneous [2,3,4,5,6]. HTLV-1 is endemic in regions of Japan, Caribbean, Central and West Africa, South America (Brazil, Colombia, Chile, and Peru), Middle East (particularly Iran), Romania, and Australo-Melanesia, and estimates indicate that 5 to 10 million individuals are infected by HTLV-1 worldwide [6,7]. However, these numbers are likely to be underestimated considering the lack of data for large areas, the increasing rate of human migration, and silent dissemination by sexual transmission in some endemic regions [8]. Indeed, HTLV-1 has been considered a re-emerging pathogen, and strategies for surveillance and prevention to reduce its incidence are imperative [9]. In turn, HTLV-2 infection is less disseminated, occurring mainly among indigenous tribes all over the American continent and among isolated groups in Africa [3,5,10]. HTLV-2 is also frequently observed in coinfection with other viruses that share the same route of transmission, such as HBV, HCV [11], and HIV [12,13], which makes HTLV-2 present in North America, Europe and Africa [12,14]. In Brazil, the risk of infection by these viruses varies considerably depending on individual factors, such as socioeconomic condition, genetic background and risk behaviors [4,11,13,15].

HTLV-1 infection is associated with a complex of neurological diseases. Approximately 2–5% of infected individuals develop an inflammatory disorder with progressive spinal cord atrophy and motor impairment known as HTLV-1-associated myelopathy/tropical spastic paraparesis, while other isolated neurological alterations, such as neurogenic bladder, peripheral neuropathy, cognitive disorders, and amyotrophic lateral sclerosis-like syndrome are less frequent [16,17]. HTLV-1 is also associated with a CD4 T-cell malignancy denominated adult T-cell leukemia/lymphoma (ATLL) [18], with inflammatory disorders, such as uveitis, arthropathy, xerosis, polymyositis, Sjogrën syndrome, and pulmonary disturbances (bronchitis, bronchiolitis, and bronchiectasis), in addition to an increased frequency of opportunistic infections, including strongyloidiasis, tuberculosis, crusted scabies, and infective dermatitis by *Staphylococcus aureus* and/or β-hemolytic *Streptococcus* [19,20,21,22,23].

The laboratory diagnosis of HTLV-1/2 infection is performed by an initial serological screening using a combination of particle agglutination test and/or distinct enzyme immunoassays (EIA), such as second and third-generation enzyme-linked immunosorbent assays (ELISA), and chemiluminescent immunoassay [24,25,26]. Thereafter, samples with a reactive, indeterminate, or discordant result in distinct serological tests are further investigated by confirmatory assays. This includes commercially available serological tests, such as Western blot (WB) and line immunoassay (LIA), and in-house molecular assays based on polymerase chain reaction (PCR), particularly the PCR with restriction fragment length polymorphism (RFLP) analysis and the real-time quantitative PCR (qPCR) using TaqMan probes. Indeed, the combination of multiple assays reduces their inherent flaws [24,25,26]. Thus, while serological tests have high sensitivity, molecular assays stand out for their specificity and lower cost [25], which is an important feature for low and middle-income countries. However, LIA is time-consuming and has an elevated cost [27], and WB shows a prevalence of indeterminate results as high as 67% of cases [28], particularly in HTLV-2 carriers [26,29,30,31] and HIV/HTLV-coinfected individuals [27,32]. To overcome this issue, it was proposed to initially test blood samples with molecular assays, which display higher specificity, and negative samples might be further tested by WB or LIA, thus improving the cost-effectiveness of HTLV-1/2 infection diagnosis [25,33,34].

Therefore, as a new alternative for the molecular diagnosis of HTLV-1/2 infection, we designed loop mediated isothermal nucleic acid amplification (LAMP) assays for these viruses. Since the LAMP method was described in the early 2000’s [35], it has been established for the detection of a growing number of human viruses of public health importance, such as hepatitis B and C viruses, HIV, herpes simplex virus, cytomegalovirus, influenza A virus, and more recently SARS-CoV-2, with equivalent to superior performance than PCR-based assays [36,37,38,39,40,41,42]. DNA amplification by LAMP is performed at a constant temperature using DNA polymerases with double-strand displacement activity and a set of three pairs of primers: (i) forward and backward inner primers (FIP and BIP, respectively), which are assembled sequences of F1c with F2, and B1c with B2, respectively; (ii) outer forward (F3) and backward (B3) primers; and (iii) loop forward (LF) and loop backward (LB) primers [35,43]. Briefly, LAMP is initiated by hybridization of the F2/B2 region of FIP/BIP to target sequences, and their extension by the DNA polymerase displaces the double-helix structure. In turn, the strand synthesized from the outer F3/B3 primers dislodges the previous strand extended from FIP/BIP. The hanging F1c/B1c region of FIP/BIP binds to their complementary sequence within the strand, originating a hairpin structure that provides a 3′-end for DNA synthesis, initiating a cyclic phase of LAMP. LF and LB primers bind to the loop of hairpin structures, therefore providing extra 3′-ends, which enhances DNA synthesis and reduces the reaction time. Thus, LAMP produces long DNA molecules containing multiple copies of the target sequence, which is visualized as a ladder pattern in agarose gel electrophoresis [35,43]. Here, we show that HTLV-1/2 LAMP assays had accuracy and sensitivity rates similar to qPCR and PCR-RFLP in distinct diagnostic scenarios, including DNA samples from HTLV-1 and HTLV-2 carriers, and individuals coinfected with HIV. Thus, the simplicity and reduced cost of LAMP tests is attractive for use in areas where laboratory infrastructure is limited.

## 2. Materials and Methods

### 2.1. Study Design and Population Data

The study was conducted at the Evandro Chagas National Institute of Infectious Diseases (INI) of the Oswaldo Cruz Foundation (FIOCRUZ), Rio de Janeiro, Brazil, with the approval of the institutional committee of ethics in research (CAAE 23369813.7.0000.5262 approved on 17 April 2014 and CAAE 98332818.9.0000.5262 approved on 7 December 2018). DNA samples were obtained from whole peripheral blood in EDTA using the Gentra Puregene blood kit (Qiagen, Gemantown, MD, USA) from individuals mono-infected with HTLV-1 (*n* = 125) and HTLV-2 (*n* = 19) followed in an open cohort between 2014 and 2017. Individuals were initially tested by ELISA (HTLV I&II Ab, Dia.Pro Diagnostic BioProbes Srl, Milano, Italy) and diagnosis was confirmed by WB (HTLV Blot 2.4, MP Biomedicals, Irvine, CA, USA) and/or PCR-RFLP. Individuals with diagnosis of HIV, HBV, and HCV infection were excluded. These DNA samples were used to determine the optimal conditions for HTLV-1/2 LAMP reactions. For the comparison between the accuracy and sensitivity of LAMP, PCR-RFLP and qPCR assays, all tests were carried out at the same time. Data on the clinical status of these patients were obtained from medical records.

The performance of HTLV-1/2 LAMP assays was also evaluated using blood DNA samples from a cohort of HTLV-1/2 carriers coinfected with HIV followed at the Adolfo Lutz Institute, São Paulo, Brazil, with the approval of the institutional committee of ethics in research on 11 April 2019 (CAAE 98332818.9.3000.0059). As previously described [34], HIV-infected individuals with reactive and indeterminate results in at least one of two ELISAs for HTLV-1/2 (Murex HTLV-I+II, DiaSorin S.p.A., Dartford, UK; Gold ELISA HTLV-I/II, REM, São Paulo, Brazil) had the diagnosis confirmed by WB (HTLV Blot 2.4, MP Biomedicals, Irvine, CA, USA), LIA (INNO-LIA HTLV-I/II, Fujirebio, Gent, Belgium), qPCR and PCR-RFLP. In total, 82 DNA samples from this cohort obtained from peripheral blood leukocytes using the MagNa Pure LC DNA Isolation kit (Roche Diagnostics, Mannheim, Germany) with the automated Extractor MagNa Pure LC 2.0 system (Roche Diagnostics, Rotkreuz, Switzerland) were tested by HTLV-1/2 LAMP assays, distributed in the following groups: HTLV-1 (*n* = 39), HTLV-2 (*n* = 35), HTLV (*n* = 3), and indeterminate (*n* = 5).

### 2.2. Design of LAMP Primers

LAMP primers were designed based on the sequences of *tax* genes of HTLV-1 ATK (GenBank J02029) and HTLV-2 Mo (GenBank M10060) strains. Primers were designed using the criteria of Parida et al. [43] with the PrimerExplorer v5 software [44]. Primer dimerization was predicted with the Multiple Primer Analyzer Tool (Thermo Fisher Scientific, Waltham, MA, USA). In addition, primer sets containing oligonucleotides with possible stable hybridization in their 3′ terminal (ΔG ≥ 5 kcal/mol) were discarded. The specificity of LAMP primers for HTLV-1 and HTLV-2 was first evaluated in silico by alignment against the HTLV-2 Mo and HTLV-1 ATK sequences, respectively, using the BioEdit v7.2.5 software [45]. Primer sets containing at least one oligonucleotide with sequence homology greater than 50% were excluded. Analysis of hairpin structures was performed with the OligoAnalyzer Tool (https://www.idtdna.com/pages/tools/oligoanalyzer), and primer sets with dissociation temperature above 50 °C were also excluded. Polymorphisms in the *tax* sequences targeted by HTLV-1/2 LAMP primers were identified by alignment between ATK and Mo strains with sequences from GenBank using the BioEdit v.7.2.5. GenBank accession numbers of HTLV-1 sequences: MF277043−MF277122, JX507077, KF242506, KX430030, KX430031, U19949, LC192536, LC192535, MH399767, MH399768, MH395864, JN887698−JN887710; HTLV-2 sequences: MF277123−MF277134, M10060, AF326583, U32875, AF139382, U32873, U32872, AF139383, AF139384, GU212854, DQ022075, L11456, Y14365.

### 2.3. HTLV-1/2 LAMP Assays

LAMP reactions were performed in a final volume of 25 μL containing 1 × ThermoPol^®^ reaction buffer, 8 U of Bst DNA polymerase Large Fragment (New England Biolabs, Ipswich, MA, USA) and 0.4 mM of deoxynucleotide triphosphates (dNTPs) (Invitrogen, Carlsbad, CA, USA). Betaine was tested at concentrations ranging from 0.6 M to 1.2 M (Sigma-Aldrich, Saint Louis, MO, USA). MgSO_4_ was evaluated at concentrations from 3 mM to 6 mM. Since the 1× ThermoPol^®^ buffer contains 2 mM of MgSO_4_, an extra 1 mM to 4 mM of MgSO_4_ was added into the reaction mixture to achieve the tested concentration. The LAMP forward inner primer (FIP) and back inner primer (BIP) were used at 40 p-moles, outer primers (F3 and B3) at 10 p-moles, and loop forward (LF) and back (LB) primers were used at 20 p-moles per reaction. Isothermal amplification was preceded by a preheating step, in which 5 μL of DNA was incubated with a mixture containing the MgSO_4_, betaine, primers and dNTPs at 95 °C for 5 min, 65 °C for 1 min, 22 °C for 5 min, and then transferred to ice. Afterwards, a mixture with Bst DNA polymerase and ThermoPol^®^ buffer was added into the tubes, and reactions were incubated at 65 °C for 1 h, followed by an enzyme inactivation step at 80 °C for 10 min.

Two-microliters of LAMP reactions were electrophoresed in 2% agarose gel (Invitrogen, Carlsbad, CA, USA) in 1× Tris-Borate-EDTA (TBE) buffer (Invitrogen, Grand Island, NY, USA) containing 0.5× GelRed™ (Biotium, Fremont, CA, USA) at 100 V for 1 h. DNA amplification was also revealed with SYBR™ Green I (Invitrogen, Carlsbad, CA, USA) added to the reaction tubes to a 100× final concentration. Results were recorded in a L-PIX UV gel imaging system (Loccus Biotecnologia, Campinas, Brazil).

### 2.4. Quantification of HTLV-1/2 Proviral Load

HTLV-1/2 proviral load was determined by qPCR with TaqMan probes in a Rotor-Gene Q 5-plex HRM Platform (Qiagen, Hilden, Germany). Peripheral blood DNA was quantified by UV spectrophotometry and diluted to 20 ng/µL. Reactions were performed in duplicates with 5 µL of diluted DNA and the Rotor-Gene Probe PCR kit (Qiagen, Hilden, Germany) in a total volume of 25 µL. A 79-bp fragment of the human *β-globin* gene was amplified for the quantification of cells, using 50 p-moles of primers (Forward, 5′-GCAAGAAAGTGCTCGGTGC-3′; reverse, 5′-TCACTCAGTGTGGCAAAGG TG-3′) and 2.5 p-moles of probe (5′-FAM-TAGTGATGGCCTGGCTCACCTGGAC-3′-TAMRA). HTLV-1/2 infected cells were detected by amplification of a 159-bp *tax* gene fragment using 15 p-moles of primers (SK43: 5′-CGGATACCCAGTCTACGTGT-3′; SK44: 5′-GAGCCGATAACGCGTCCATCG-3′) and 5 p-moles of probe (SK45: 5′-FAM-ACGCCCTA CTGGCCACCTGTC-3′-TAMRA). Amplification was carried out in the following conditions: enzyme activation at 95 °C for 5 min, 45 cycles of denaturation at 95 °C for 5 s and hybridization/extension at 60 °C for 15 s, and fluorescence detection at the end of each cycle. The corresponding number of cells in the reaction was determined in a standard curve constructed with two-fold dilutions of human DNA (Promega, Madison, WI, USA), ranging from 12.5 ng to 400 ng, and the number of infected cells was calculated with a standard curve constructed with two-fold dilutions of DNA from TARL-2 cell line (RRID: CVCL_0557) (from 3.125 ng to 200 ng per reaction), which is a T-cell lineage carrying a single copy of the HTLV-1 provirus per cell [46]. Standard curves showed R^2^ >0.998, and the HTLV-1/2 proviral load was defined as the percentage of infected cells in peripheral blood leukocytes with the formula: [(*tax* copies)/(copies of *β-globin*/2)] × 100.

### 2.5. PCR-RFLP for HTLV-1/2

PCR was performed with the Platinum™ Taq DNA Polymerase (Invitrogen, São Paulo, Brazil) and 15 p-moles of SK43 and SK44 primers. Reactions were carried out with 5 µL of DNA in a total volume of 50 µL containing: 1× PCR buffer, 2 mM of MgCl_2_, 0.2 mM of dNTPs and 1.25 U of Platinum™ Taq DNA polymerase. PCR was performed in the following conditions: enzyme heat activation at 95 °C for 3 min, 45 cycles of denaturation at 94 °C for 30 s, hybridization at 60 °C for 30 s and extension at 72 °C for 30 s, followed by a final extension step at 72 °C for 5 min. Subsequently, RFLP was performed by digestion of 20 µL of PCR products with 20 U of TaqI restriction enzyme (Promega, Madison, WI, USA) at 65 °C for 4 h, following the manufacturer’s instructions. The result was revealed by electrophoresis in 2% agarose gel in 1× TBE buffer with 0.5× GelRed™ at 100 V for 90 min.

### 2.6. Statistical Analysis

The accuracy and sensitivity of HTLV-1/2 LAMP assays were calculated in 2 × 2 contingency tables using the R software version 3.6.1. The definitive diagnosis of HTLV-1/2 infection was determined by a positive result in any of the confirmatory assays performed (WB, INNO-LIA, qPCR or PCR-RFLP). Samples with an indeterminate status were considered negative in the analysis. The overall accuracy and sensitivity rates were calculated with results from all patients included, regardless of their original cohort, and also separately for groups of individuals with HTLV-1/2 single infection and coinfected with HIV. The 95% confidence intervals (95% CI) were calculated by the Clopper-Pearson method using the DeskTools package for R software. The agreement between results from the molecular tests was performed using Cochran’s Q-test, and comparisons with *p* < 0.05 were tested by post-hoc pairwise McNemar with Bonferroni adjustment. Differences with a *p*-value of <0.05 were considered significant.

## 3. Results

### 3.1. Genetic Polymorphisms within LAMP Primers for HTLV-1/2

**Figure 1:**
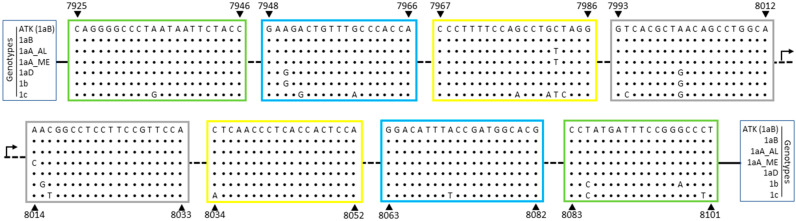
HTLV-1 *tax* sequence recognized by LAMP primers. HTLV-1 genome sequence corresponding to LAMP primers designed based on the ATK strain (GenBank J02029, which belongs in Table 1; aB), were aligned with representative sequences from other HTLV-1 genotypes: 1aA_LA, Cosmopolitan Transcontinental, Latin American cluster; 1aA_ME, Cosmopolitan Transcontinental, Middle East cluster; 1aD, North Africa; 1b, Africa; and 1c, Melanesia. The nucleotide positions are related to the ATK strain (indicated by arrows) and the regions for primer hybridization are highlighted in boxes: green: F3/B3; blue: F2/B2; yellow: LF/LB; gray: F1c/B1c.

HTLV-1/2 LAMP primers (Table 1) were designed based on the *tax* sequences from the HTLV-1 ATK and the HTLV-2 Mo strains, which belong to the HTLV-1 Cosmopolitan Japanese (1aB) and the HTLV-2a genotypes, respectively. Sequence alignment between HTLV-1 primers and HTLV-1aB isolates showed no nucleotide substitutions within target sequences (Figure 1). Among Brazilian isolates, which are mainly of the Latin American cluster of the Cosmopolitan Transcontinental genotype (1aA), a C7982T substitution was observed within the 5′ region of the LF primer (Figure 1). This polymorphism was also seen in the HTLV-1aA Middle East cluster, in addition to a A8014C substitution at the BIP 5′-terminal (Figure 1, B1c region). The Cosmopolitan North African (1aD) and the African (1b) genotypes showed 2 and 5 nucleotide substitutions, respectively, in a total of 159 nucleotides covered by primers (Figure 1). In turn, the Melanesian genotype (1c), which is the most divergent HTLV-1 genotype, had a total of 14 nucleotide substitutions (Figure 1).

**Figure 2:**
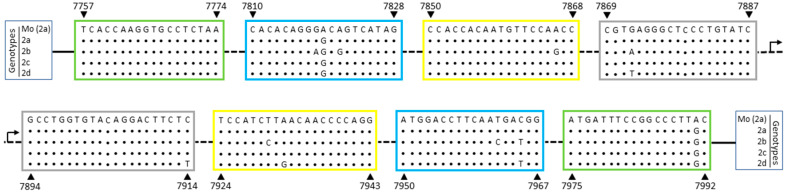
HTLV-2 *tax* sequence recognized by LAMP primers. HTLV-2 genome sequence corresponding to LAMP primers designed based on the Mo strain (GenBank M10060), HTLV-2a genotype, were aligned with representative sequences of all four HTLV-2 genotypes: 2a, 2b, 2c, and 2d. The nucleotide positions are related to the Mo strain (indicated by arrows) and the regions for primer hybridization are highlighted in boxes: green: F3/B3; blue: F2/B2; yellow: LF/LB; gray: F1c/B1c.

Sequence analysis of LAMP primers for HTLV-2 showed that isolates of all HTLV-2 genotypes (a, b, c, and d) had 2 nucleotide polymorphisms, A7819G and A7991G, respectively located within the regions of F2 and B3 primers (Figure 2). Thus, the G polymorphism was chosen for these positions in order to adjust the primer sequences (Table 1). Thereafter, only HTLV-2b and 2d genotypes presented genetic polymorphisms, with respectively 7 and 4 nucleotide substitutions in a total of 152 nucleotides recognized by primers (Figure 2).

### 3.2. Optimization and Target Specificity of HTLV-1/2 LAMP Reactions

**Figure 3:**
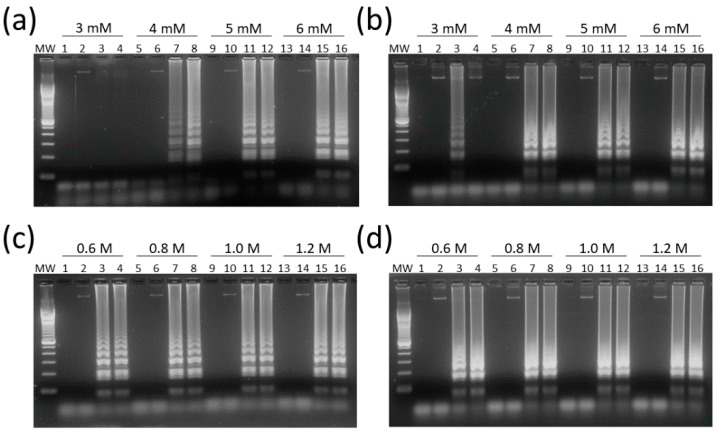
Optimization of magnesium and betaine concentrations in HTLV-1/2 LAMP assays. (**a**) HTLV-1 and (**b**) HTLV-2 LAMP assays were performed with MgSO_4_ at concentrations of 3–6 mM. Betaine was tested at concentrations of 0.6 M to 1.2 M in assays for (**c**) HTLV-1 and (**d**) HTLV-2. Reactions were performed with 5 µL of a 20 ng/µL DNA dilution, and 2 µL of LAMP products were submitted to electrophoresis in 2% agarose gel in 1× Tris-Borate-EDTA (TBE) with 0.5× GelRed at 100 V for 1 h. MW, 100-bp DNA molecular weight. Lanes 1, 5, 9, and 13: no DNA template control (water). Lanes 2, 6, 10, and 14: HTLV-1/2 negative samples. Lanes 3, 4, 7, 8, 11, 12, 15, and 16: DNA samples positive for (**a**,**c**) HTLV-1 and (**b**,**d**) HTLV-2.

HTLV-1/2 LAMP assays were adjusted for optimal concentrations of Mg^2+^, a DNA polymerase cofactor, and betaine, which reduces the formation of secondary structures in guanine-cytosine (GC)-rich regions. MgSO_4_ at 6 mM had the best result in LAMP reactions for both HTLV-1 and HTLV-2 (Figure 3a,b, respectively).

**Figure 4:**
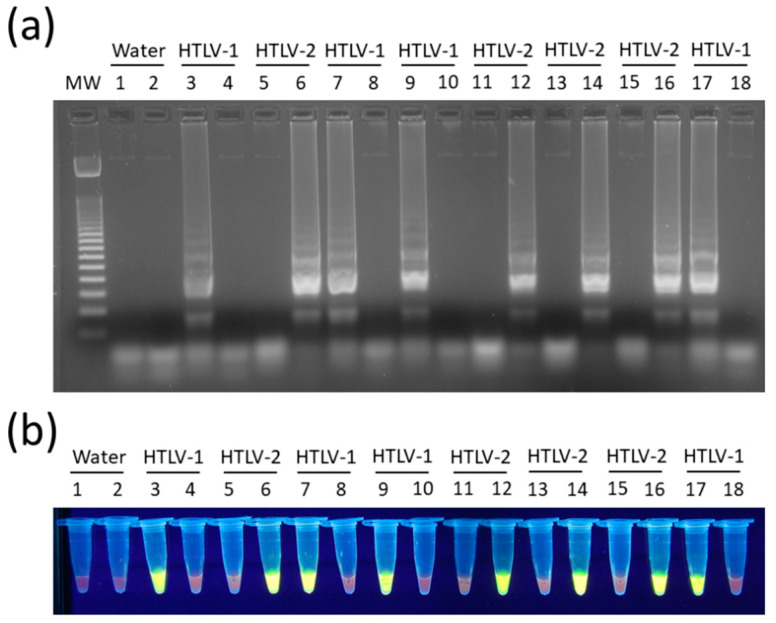
Specificity of HTLV-1/2 LAMP assays. Reactions were performed with DNA samples positive for HTLV-1 or HTLV-2, where indicated. LAMP products were revealed by (**a**) electrophoresis in 2% agarose gel in 1× TBE with 0.5× GelRed, and (**b**) with SYBR^TM^ Green I added to tubes to a 100× final concentration. MW, 100-bp DNA molecular weight; Water, no template control; LAMP assays specific for HTLV-1 (odd lanes) or HTLV-2 (even lanes).

In turn, the concentration of 0.8 M of betaine was chosen since no difference was observed between LAMP reactions containing 0.6 M to 1.2 M in assays for both HTLV-1 (Figure 3c) and HTLV-2 (Figure 3d). In addition, LAMP primers for HTLV-1/2 were specific for their respective targets, showing no cross-reactivity, and DNA amplification could also be shown by addition of SYBR Green I dye into tubes (Figure 4).

### 3.3. Overall Accuracy and Sensitivity of HTLV-1/2 LAMP Assays

A total of 226 DNA samples from individuals with laboratory data for the diagnosis of HTLV-1/2 infection from two distinct cohorts were tested by HTLV-1/2 LAMP assays. This included patients mono-infected by HTLV-1 (*n* = 125) and HTLV-2 (*n* = 19), and with HIV/HTLV-1 (*n* = 39) and HIV/HTLV-2 (*n* = 35) coinfections, in addition to HIV carriers coinfected with non-typed HTLV (*n* = 3) or with HTLV indeterminate status (*n* = 5). As shown in Table 2, similar rates were obtained for the overall accuracy of LAMP (95% CI 74.8–85.5%), qPCR (95% CI 69.5–81.1%), and PCR-RFLP (95% CI 79.5–89.3%) in the confirmatory diagnosis of HTLV-1/2 infection, as well as for their sensitivity. No difference was seen in the accuracy rates between LAMP for HTLV-1 (95% CI 87.7–95.2%) and HTLV-2 (95% CI 85.1–93.4%) (Table 2). However, the sensitivity of LAMP for HTLV-1 (95% CI 83.2–93.4%) was significantly greater than values of LAMP for HTLV-2 (95% CI 43.2–70.8%) (Table 2). Indeed, this discrepancy was also seen in the results from qPCR and PCR-RFLP assays (Table 2). Meanwhile, LAMP, qPCR and PCR-RFLP showed better results in detecting HTLV-1/2 proviruses in DNA samples from HTLV-1/2 mono-infected individuals in comparison with patients coinfected with HIV (Table 2), probably as a result of lower CD4 T-cell counts in these patients. In general, LAMP had a performance similar to or superior compared to the other molecular assays. Moreover, LAMP did not display discrepant results in comparison with PCR-RFLP in all populations evaluated (Table 3), while a significant difference was observed between LAMP and qPCR among mono-infected HTLV-1/2 patients (Table 3, *p* < 0.003), possibly as a result of the higher accuracy of LAMP.

## 4. Discussion

The main objective of this study was to develop a LAMP assay for the confirmatory diagnosis of HTLV-1/2 infection through detection of proviruses in blood DNA samples. Thus, sets of primers were designed for *tax* gene regions with low homology (<50%) between HTLV-1 and HTLV-2 to eliminate the possibility of cross-reaction. In addition, conserved regions of the *tax* gene of each virus were selected, and regions with frequent genetic polymorphisms described for distinct genotypes were avoided [47,48,49]. The formation of secondary structures, such as primer-dimers and hairpins, which can occur in long oligonucleotides such as FIP and BIP, was also evaluated to prevent loss in LAMP performance [50] and/or the generation of false-positive results. Nonetheless, LAMP assays require a set of three pairs of primers presenting specific features. The fact that HTLV-1/2 proviruses have approximately 9 kb restricted the definition of suitable LAMP target sequences. It is noteworthy that nucleotide substitutions can lower the affinity between primers and their target sequences. However, LAMP is able to amplify target sequences without loss in activity even in the presence of 12 to 14 nucleotide substitutions [51].

The HTLV-1 *tax* sequence recognized by LAMP primers had only one nucleotide substitution between the Cosmopolitan Transcontinental (1aA) and Japanese (1aB) genotypes, which are the most prevalent in Brazil and Japan, respectively [6]. In contrast, the main difference was seen against the Melanesian/Australian HTLV-1 genotype (1c), which is the most divergent [52,53], and even presents high diversity within the same genotype [54,55]. Therefore, although HTLV-1c prevalence varies greatly in Melanesia, from 0.6% in Ni-Vanuatu and New Caledonia ethnicities [54,56] to 33.6% in indigenous population in Central Australia [7], this genotype is not found elsewhere [6,14,53]. Thus, it would be interesting to have the LAMP assays described here tested among these populations, since this genotype was not present in our cohort [47]. In turn, HTLV-2 provirus region recognized by LAMP primers showed low divergence, with less than seven genetic polymorphisms among all HTLV-2 genotypes.

Previously, Campos et al. [34] showed that WB had indeterminate results in 22.2% of the samples (26 of 117). Six in 17 of these samples were positive by LAMP (five for HTLV-2 and one for HTLV-1) (Table S1). This corroborates the observation that half of the samples with indeterminate results in the WB are positive by PCR [57]. This issue has been associated with low antigenic stimulation due to the selection of defective proviruses [58,59], which in turn prolongs the time for seroconversion [32]. Serological and molecular follow-up of individuals with indeterminate WB results showed that seroconversion in some cases might take several years [60,61]. Furthermore, a study with macaques infected with the simian T-lymphotropic virus 1 associated the late seroconversion with the presence of polymorphisms in the *pol* and *rex* genes [62]. Moreover, 10 of 13 samples from HTLV-1/2 mono-infected patients, indeterminate or positive for both HTLV-1 and HTLV-2 by WB, were positive for only HTLV-1 by LAMP and qPCR or PCR-RFLP (Table S2), indicating false-positive results for HTLV-2. In this population, one patient was also diagnosed with HTLV-2 by WB, which was later confirmed as HTLV-1. Therefore, molecular assays are useful in solving limitations of confirmatory serological tests.

It has been shown that *tax* expression is lost in approximately half of ATLL clones, and this process is associated with 5′-long terminal repeats (LTR) deletion or methylation, or non-sense mutations, deletions or insertions within the *tax* gene [63,64]. However, the population of HTLV-1-infected cells is heterogeneous, and it is constituted by tens of thousands of distinct T-cell clones identified by their unique provirus integration sites into the host cell DNA [65]. Thus, genetic alterations accumulated during HTLV-1-induced leukemogenesis are not widely distributed among distinct infected T-cell clones [18]. ATLL is a malignancy of mature CD4 T-cells, but HTLV-1 can infect various cell types, including CD8 T-cells, B cells, and dendritic cells, although in a lower extent [66]. Therefore, although genetic aberrations in HTLV-1 provirus may abrogate *tax* expression in ATLL cells or hinder the detection of such clonal populations by molecular assays targeting *tax* sequences, interference of this process with the detection of untransformed cells in ATLL patients by LAMP or any PCR-based assay seems unlikely. However, deletions and insertions in *tax* might indeed be a limitation to molecular assays targeting this gene. Conversely, possible pitfalls of LAMP in the diagnosis of HTLV-1 infection can be improved by performing serological tests in samples with a negative result.

The sensitivity of the HTLV-1 LAMP assay was invariably higher than the test for HTLV-2, as also observed in PCR-based tests. This was associated with low proviral load commonly seen in HTLV-2 infection in comparison with HTLV-1 [67,68,69], and the LAMP method unfortunately did not improve this limitation of molecular assays for the confirmatory diagnosis of HTLV-2 infection. Moreover, SK43 and SK44 primers commonly used worldwide present two mismatches each on the target sequence of HTLV-2 [70]. This was evident in qPCR performed with these primers and the SK45 probe in the cohort of mono-infected HTLV-1/2 individuals. Conversely, consistently high values of overall accuracy of molecular assays were observed in the detection of each virus regardless of the population studied (Tables S3 and S4). This reflected the higher specificity of molecular assays compared with serological tests for the confirmatory diagnosis of HTLV-1/2 infection [24,34]. However, it was not possible to define the specificity rates of LAMP assays due to the study design and absence of false positive results.

Thus, the use of LAMP assays described here prior to confirmatory serological tests would reduce costs of HTLV-1/2 diagnosis. HTLV-1/2 LAMP assays are a reliable alternative to PCR-based tests, reducing the time for diagnosis in comparison with PCR-RFLP, which depends on amplification, digestion with endonucleases, and agarose gel electrophoresis to reveal the result, and simplifying the laboratory infrastructure compared to qPCR. LAMP has the advantage of being carried out in a conventional thermocycler, or even in a water bath or thermal block. In addition, LAMP results can also be revealed by SYBR Green I by pre-loading the dye in the inner side of microtube caps before the reaction, since it inhibits DNA amplification by *Bst* DNA polymerases [7], or using DNA dyes such as SYTO9, SYTO13 and SYTO16 directly in the reaction mixture [71]. This abrogates the need for opening tubes after the reaction, thus minimizing the chance of amplicon contamination.

## Figures and Tables

**Table 1:** Loop-Mediated Isothermal Amplification (LAMP) primers for human-T lymphotropic viruses HTLV-1 and HTLV-2.

Target	Primers ^1^	Sequence (5′→3′) ^2^	Positions ^2,3^
HTLV-1	FIP-1	5′-**tgccaggctgttagcgtgac**gaagactgtttgcccacca-3′	**(F1c: 8012–7993)** (F2: 7948**–**7966)
	BIP-1	5′-**aacggcctccttccgttcca**cgtgccatcggtaaatgtcc-3′	**(B1c: 8014–8033)** (B2: 8082**–8**063)
	F3-1	5′-caggggccctaataattctacc-3′	7925–7946
	B3-1	5′-agggcccggaaatcatagg-3′	8101–8083
	LF-1	5′-cctagcaggctggaaaaggg-3′	7986–7967
	LB-1	5′-ctcaaccctcaccactcca-3′	8034–8052
HTLV-2	FIP-2	5′-**gatacagggagccctcacg**cacacaggggcagtcatag-3′	**(F1c: 7887–7869)** (F2: 7810–7828)
	BIP-2	5′-**gcctggtgtacaggacttctc**ccgtcattgaaggtccat-3′	**(B1c: 7894–7914)** (B2: 7967–7950)
	F3-2	5′-tcaccaaggtgcctctaa-3′	7757–7774
	B3-2	5′-gcaagggccggaaatcat-3′	7992–7975
	LF-2	5′-ggttggaacattgtggtgg-3′	7868–7850
	LB-2	5′-tccatcttaacaaccccagg-3′	7924–7943

^1^ Forward inner primers (FIP) and backward inner primers (BIP) are assembled sequences of F1c + F2 and B1c + B2, respectively. ^2^ F1c/B1c regions are in bold, and F2/B2 sequences are underlined. ^3^ Nucleotide positions according to HTLV-1 ATK (J02029) and HTLV-2 Mo (M10060) strains.

**Table 2:** Accuracy and sensitivity of LAMP, qPCR and PCR-RFLP assays for HTLV-1/2.

Groups		LAMP		qPCR		PCR-RFLP	
	*n*	Accuracy	Sensitivity	Accuracy	Sensitivity	Accuracy	Sensitivity
Total ^1^	226	80.5	80.1	75.7	75.1	84.9	84.6
		(74.8–85.5)	(74.2–85.2)	(69.5–81.1)	(68.9–80.7)	(79.5–89.3)	(79.1–90.0)
HTLV-1	164	92.0	89.0	88.1	83.5	94.7	93.9
		(87.7–95.2)	(83.2–93.4)	(83.1–92.0)	(77.0–88.9)	(90.9–97.2)	(89.0–97.0)
HTLV-2	54	89.8	57.4	88.9	53.7	89.8	57.4
		(85.1–93.4)	(43.2–70.8)	(84.1–92.7)	(40.0–67.4)	(85.1–93.4)	(43.2–70.8)
HTLV-1/2 mono-infected	144	88.9	88.9	79.9	79.9	93.1	93.1
		(82.6–93.5)	(82.6–93.5)	(72.4–86.1)	(72.4–86.1)	(87.6–96.6)	(87.6–96.6)
HIV-coinfected	82	65.9	63.6	68.3	66.2	70.4	68.4
		(54.6–76.0)	(51.9–74.3)	(57.1–78.1)	(54.6–76.6)	(59.2–80.0)	(56.8–78.6)

The overall accuracy and sensitivity of HTLV-1/2 LAMP, real-time quantitative PCR (qPCR), and PCR-Restriction Fragment Length Polymorphism (PCR-RFLP) assays are shown in percentage. 95% confidence intervals are presented inside parenthesis. ^1^ The total number of patients includes non-typed HTLV (*n* = 5) and indeterminate (*n* = 3) results.

**Table 3:** Agreement between molecular assays in the diagnosis of HTLV-1/2 infection.

	Cochran’s Q-Test	Post-Hoc McNemar
All patients (*n* = 226)		
LAMP × PCR-RFLP	14.4 (<0.001)	2.8 (0.284)
LAMP × qPCR		4.5 (0.103)
PCR-RFLP × qPCR		10.5 (0.004)
HTLV-1/2 mono-infected (*n* = 144)		
LAMP × PCR-RFLP	21.8 (<0.001)	2.0 (0.471)
LAMP × qPCR		11.3 (0.003)
PCR-RFLP × qPCR		19 (<0.001)
HIV-coinfected (*n* = 82)		
LAMP × PCR-RFLP	1.2 (0.549)	N.A.
LAMP × qPCR		N.A.
PCR-RFLP × qPCR		N.A.

The agreement between the molecular assays was evaluated with Cochran’s *Q*-test with results from all patients, or groups of HTLV-1/2 mono-infected and HIV-coinfected separately. When differences in Cochran’s *Q*-test were significant (*p* < 0.05), post-hoc McNemar with Bonferroni adjustment was used for pairwise comparisons between LAMP, real-time quantitative PCR (qPCR), and PCR-Restriction Fragment Length Polymorphism (PCR-RFLP). N.A., not applicable.

## Supplementary Materials

The following are available online at https://www.mdpi.com/1999-4915/12/9/981/s1. Table S1: Data set from HIV carriers coinfected with HTLV-1/2, Table S2: Overall accuracy and sensitivity of LAMP, qPCR and PCR-RFLP assays in individuals mono-infected by HTLV-1/2, Table S3: Overall accuracy and sensitivity of LAMP, qPCR and PCR-RFLP assays in HIV carriers coinfected with HTLV-1/2, Table S4: Data set from HTLV-1/2 mono-infected patients.
