# Supplementary material for: Loop-Mediated Isothermal Amplification (LAMP) Assay for Rapid and Accurate Confirmatory Diagnosis of HTLV-1/2 Infection
Source: Viruses. 2020 Sep 4;12(9):981. doi: 10.3390/v12090981 (PMC7552020; doi:10.3390/v12090981)
Supplement: Supplementary file 1 [file viruses-12-00981-s001.pdf]

Table S1: Data set from HIV carriers coinfected with HTLV-1/2.

ID	qPCR	PCR-RFLP	Definitive result[a]	LAMP	ID	qPCR	PCR-RFLP	Definitive result[a]	LAMP
**1**	**Neg**	HTLV-2	HTLV-2	HTLV-2	**49**	HTLV-1	HTLV-1	HTLV-1	HTLV-1
**2**	**Neg**	**Neg**	HTLV	**Neg**	**50**	HTLV-2	**Neg**	HTLV-2	HTLV-2
**3**	HTLV-1	HTLV-1	HTLV-1	HTLV-1	**66**	HTLV-1	HTLV-1	HTLV-1	HTLV-1
**4**	**Neg**	HTLV-1	HTLV-1	**Neg**	**67**	**Neg**	**Neg**	HTLV-2	**Neg**
**5**	**Neg**	**Neg**	HTLV-2	**Neg**	**68**	HTLV-1	HTLV-1	HTLV-1	HTLV-1
**6**	**Neg**	**Neg**	HTLV-1	**Neg**	**71**	HTLV-2	HTLV-2	HTLV-2	HTLV-2
**7**	**Neg**	HTLV-1	HTLV-1	**Neg**	**72**	HTLV-1	HTLV-1	HTLV-1	**Neg**
**8**	**Neg**	**Neg**	HTLV-2	**Neg**	**74**	**Neg**	**Neg**	HTLV-1	**Neg**
**9**	HTLV-1	HTLV-1	HTLV-1	HTLV-1	**76**	HTLV-2	**Neg**	HTLV-2	**Neg**
**10**	HTLV-1	HTLV-1	HTLV-1	HTLV-1	**79**	**Neg**	**Neg**	HTLV	**Neg**
**11**	**Neg**	**Neg**	HTLV-1	**Neg**	**81**	HTLV-2	HTLV-2	HTLV-2	HTLV-2
**12**	HTLV-2	**Neg**	HTLV-2	**Neg**	**82**	HTLV-2	HTLV-2	HTLV-2	HTLV-2
**13**	HTLV-1	HTLV-1	HTLV-1	HTLV-1	**83**	**Neg**	HTLV-2	HTLV-2	**Neg**
**14**	**Neg**	**Neg**	HTLV-2	**Neg**	**84**	HTLV-1	HTLV-1	HTLV-1	HTLV-1
**15**	HTLV-1	**Neg**	HTLV-1	HTLV-1	**88**	HTLV-1	HTLV-1	HTLV-1	HTLV-1
**16**	HTLV-1	HTLV-1	HTLV-1	HTLV-1	**89**	HTLV-2	HTLV-2	HTLV-2	HTLV-2
**17**	HTLV-2	HTLV-2	HTLV-2	HTLV-2	**90**	HTLV-2	HTLV-2	HTLV-2	HTLV-2
**18**	**Neg**	**Neg**	HTLV-2	**Neg**	**91**	HTLV-1	HTLV-1	HTLV-1	HTLV-1
**19**	**Neg**	**Neg**	HTLV-2	**Neg**	**93**	HTLV-2	**Neg**	HTLV-2	**Neg**
**20**	**Neg**	HTLV-1	HTLV-1	HTLV-1	**95**	HTLV-2	HTLV-2	HTLV-2	HTLV-2
**21**	**Neg**	HTLV-1	HTLV-1	HTLV-1	**96**	HTLV-1	HTLV-1	HTLV-1	HTLV-1
**22**	HTLV-1	HTLV-1	HTLV-1	HTLV-1	**97**	HTLV-1	HTLV-1	HTLV-1	HTLV-1
**23**	**Neg**	**Neg**	HTLV	**Neg**	**98**	HTLV-1	HTLV-1	HTLV-1	HTLV-1
**24**	HTLV-1	**Neg**	HTLV-1	HTLV-1	**100**	HTLV-1	NP	HTLV-1	HTLV-1
**25**	HTLV-2	**Neg**	HTLV-2	**Neg**	**101**	HTLV-2	HTLV-2	HTLV-2	HTLV-2
**26**	**Neg**	**Neg**	HTLV-1	**Neg**	**102**	**Neg**	HTLV-2	HTLV-2	**Neg**
**27**	HTLV-1	HTLV-1	HTLV-1	HTLV-1	**103**	HTLV-2	HTLV-2	HTLV-2	HTLV-2
**28**	**Neg**	**Neg**	HTLV-2	**Neg**	**104**	**Neg**	**Neg**	HTLV-2	**Neg**
**29**	HTLV-1	HTLV-1	HTLV-1	HTLV-1	**105**	HTLV-1	HTLV-1	HTLV-1	HTLV-1
**30**	HTLV-1	HTLV-1	HTLV-1	HTLV-1	**106**	HTLV-2	HTLV-2	HTLV-2	HTLV-2
**31**	HTLV-1	HTLV-1	HTLV-1	HTLV-1	**107**	HTLV-2	HTLV-2	HTLV-2	HTLV-2
**32**	HTLV-1	HTLV-1	HTLV-1	HTLV-1	**112**	**Neg**	**Neg**	IND	**Neg**
**33**	HTLV-1	HTLV-1	HTLV-1	HTLV-1	**114**	**Neg**	**Neg**	IND	**Neg**
**34**	**Neg**	HTLV-1	HTLV-1	HTLV-1	**115**	**Neg**	**Neg**	IND	**Neg**
**35**	HTLV-1	HTLV-1	HTLV-1	HTLV-1	**116**	**Neg**	**Neg**	IND	**Neg**
**36**	HTLV-1	**Neg**	HTLV-1	**Neg**	**117**	**Neg**	**Neg**	IND	**Neg**
**37**	**Neg**	**Neg**	HTLV-2	**Neg**	**118**	–	–	**Neg**	**Neg**
**38**	HTLV-2	HTLV-2	HTLV-2	HTLV-2	**119**	–	–	**Neg**	**Neg**
**39**	HTLV-1	HTLV-1	HTLV-1	HTLV-1	**120**	–	–	**Neg**	**Neg**
**40**	HTLV-2	HTLV-2	HTLV-2	HTLV-2	**121**	–	–	**Neg**	**Neg**
**41**	HTLV-2	HTLV-2	HTLV-2	HTLV-2	**122**	–	–	**Neg**	**Neg**
**42**	HTLV-2	HTLV-2	HTLV-2	HTLV-2	**123**	–	–	**Neg**	**Neg**
**43**	HTLV-2	HTLV-2	HTLV-2	HTLV-2	**124**	–	–	**Neg**	**Neg**
**44**	HTLV-2	**HTLV-1**	HTLV-2	**Neg**	**125**	–	–	**Neg**	**Neg**
**46**	**Neg**	HTLV-1	HTLV-1	HTLV-1	**126**	–	–	**Neg**	**Neg**
**48**	**Neg**	**HTLV-1**	HTLV-2	**Neg**	**127**	–	–	**Neg**	**Neg**

Notes. HIV carriers were investigated for coinfection with HTLV-1/2 by serological screening using two ELISA and reactive and indeterminate results were confirmed by INNO-LIA, Western blot, real-time

PCR (qPCR), and PCR-restriction fragment length polymorphism (PCR-RFLP), as previously published by Campos et al. [27].

[a] The definitive result was determined by a positive result in any of the confirmatory tests. DNA samples from this study were evaluated by loop-mediated isothermal amplification (LAMP) assays, and samples used were indicated with the same identification (ID) numbers from the original work of Campos et al. [27]. IND, indeterminate.

Table S2: Data set from HTLV-1/2 monoinfected patients.

ID	WB	qPCR	PCR-RFLP	Definitive result[a]	LAMP	ID	WB	qPCR	PCR-RFLP	Definitive result[a]	LAMP
**01**	HTLV-1	HTLV-1	HTLV-1	HTLV-1	HTLV-1	**37**	n.d.	HTLV-1	HTLV-1	HTLV-1	HTLV-1
**02**	HTLV-1	HTLV-1	HTLV-1	HTLV-1	HTLV-1	**38**	HTLV-1	HTLV-1	HTLV-1	HTLV-1	HTLV-1
**03**	HTLV-1	HTLV-1	HTLV-1	HTLV-1	HTLV-1	**39**	n.d.	HTLV-1	HTLV-1	HTLV-1	HTLV-1
**04**	n.d.	HTLV-1	HTLV-1	HTLV-1	HTLV-1	**40**	HTLV-1	HTLV-1	HTLV-1	HTLV-1	HTLV-1
**05**	n.d.	HTLV-1	HTLV-1	HTLV-1	HTLV-1	**41**	n.d.	HTLV-1	HTLV-1	HTLV-1	HTLV-1
**06**	HTLV-1	HTLV-1	HTLV-1	HTLV-1	HTLV-1	**42**	HTLV-1	HTLV-1	HTLV-1	HTLV-1	HTLV-1
**07**	HTLV-1	HTLV-1	HTLV-1	HTLV-1	HTLV-1	**43**	HTLV-1	HTLV-1	HTLV-1	HTLV-1	HTLV-1
**08**	HTLV-1	HTLV-1	HTLV-1	HTLV-1	HTLV-1	**44**	HTLV-1	HTLV-1	HTLV-1	HTLV-1	HTLV-1
**09**	HTLV-1	HTLV-1	HTLV-1	HTLV-1	HTLV-1	**45**	HTLV-1	HTLV-1	HTLV-1	HTLV-1	HTLV-1
**10**	n.d.	HTLV-1	HTLV-1	HTLV-1	HTLV-1	**46**	HTLV-1	HTLV-1	HTLV-1	HTLV-1	HTLV-1
**11**	n.d.	HTLV-1	HTLV-1	HTLV-1	HTLV-1	**47**	HTLV-1	HTLV-1	HTLV-1	HTLV-1	HTLV-1
**12**	IND	HTLV-1	HTLV-1	HTLV-1	HTLV-1	**48**	HTLV-1	HTLV-1	HTLV-1	HTLV-1	HTLV-1
**13**	HTLV-1	HTLV-1	HTLV-1	HTLV-1	HTLV-1	**49**	HTLV-1	HTLV-1	HTLV-1	HTLV-1	HTLV-1
**14**	HTLV-1	HTLV-1	HTLV-1	HTLV-1	HTLV-1	**50**	HTLV-1	HTLV-1	HTLV-1	HTLV-1	HTLV-1
**15**	HTLV-1	HTLV-1	HTLV-1	HTLV-1	HTLV-1	**51**	HTLV-1	HTLV-1	HTLV-1	HTLV-1	HTLV-1
**16**	HTLV-1	HTLV-1	HTLV-1	HTLV-1	HTLV-1	**52**	HTLV-1	HTLV-1	HTLV-1	HTLV-1	HTLV-1
**17**	n.d.	HTLV-1	HTLV-1	HTLV-1	HTLV-1	**53**	HTLV-1	HTLV-1	HTLV-1	HTLV-1	HTLV-1
**18**	IND	HTLV-1	HTLV-1	HTLV-1	HTLV-1	**54**	HTLV-1	HTLV-1	HTLV-1	HTLV-1	HTLV-1
**19**	HTLV-1	HTLV-1	HTLV-1	HTLV-1	HTLV-1	**55**	HTLV-1	HTLV-1	HTLV-1	HTLV-1	HTLV-1
**20**	HTLV-1	HTLV-1	HTLV-1	HTLV-1	HTLV-1	**56**	HTLV-1+2	HTLV-1	HTLV-1	HTLV-1	HTLV-1
**21**	n.d.	HTLV-1	HTLV-1	HTLV-1	HTLV-1	**57**	n.d.	HTLV-1	HTLV-1	HTLV-1	HTLV-1
**22**	HTLV-1	HTLV-1	HTLV-1	HTLV-1	HTLV-1	**58**	HTLV-1	HTLV-1	HTLV-1	HTLV-1	HTLV-1
**23**	HTLV-1	HTLV-1	HTLV-1	HTLV-1	HTLV-1	**59**	HTLV-1	HTLV-1	HTLV-1	HTLV-1	HTLV-1
**24**	HTLV-1	HTLV-1	HTLV-1	HTLV-1	HTLV-1	**60**	HTLV-1	HTLV-1	HTLV-1	HTLV-1	HTLV-1
**25**	HTLV-1	HTLV-1	HTLV-1	HTLV-1	HTLV-1	**61**	HTLV-1	HTLV-1	HTLV-1	HTLV-1	HTLV-1
**26**	n.d.	HTLV-1	HTLV-1	HTLV-1	HTLV-1	**62**	HTLV-1+2	HTLV-1	HTLV-1	HTLV-1	HTLV-1
**27**	n.d.	HTLV-1	HTLV-1	HTLV-1	HTLV-1	**63**	HTLV-1	HTLV-1	HTLV-1	HTLV-1	HTLV-1
**28**	HTLV-1+2	HTLV-1	HTLV-1	HTLV-1	HTLV-1	**64**	HTLV-1	HTLV-1	HTLV-1	HTLV-1	HTLV-1
**29**	HTLV-1	HTLV-1	HTLV-1	HTLV-1	HTLV-1	**65**	n.d.	HTLV-1	HTLV-1	HTLV-1	HTLV-1
**30**	HTLV-1+2	HTLV-1	HTLV-1	HTLV-1	HTLV-1	**66**	n.d.	HTLV-1	HTLV-1	HTLV-1	HTLV-1
**31**	HTLV-1	HTLV-1	HTLV-1	HTLV-1	HTLV-1	**67**	n.d.	HTLV-1	HTLV-1	HTLV-1	HTLV-1
**32**	n.d.	HTLV-1	HTLV-1	HTLV-1	HTLV-1	**68**	HTLV-1+2	HTLV-1	HTLV-1	HTLV-1	HTLV-1
**33**	HTLV-1	HTLV-1	HTLV-1	HTLV-1	HTLV-1	**69**	HTLV-1	HTLV-1	HTLV-1	HTLV-1	HTLV-1
**34**	HTLV-1+2	HTLV-1	HTLV-1	HTLV-1	HTLV-1	**70**	HTLV-1	HTLV-1	HTLV-1	HTLV-1	HTLV-1
**35**	n.d.	HTLV-1	HTLV-1	HTLV-1	HTLV-1	**71**	n.d.	HTLV-1	HTLV-1	HTLV-1	HTLV-1
**36**	n.d.	HTLV-1	HTLV-1	HTLV-1	HTLV-1	**72**	HTLV-1	HTLV-1	HTLV-1	HTLV-1	HTLV-1

Table S2 (continued)

ID	WB	qPCR	PCR-RFLP	Definitive result[a]	LAMP	ID	WB	qPCR	PCR-RFLP	Definitive result[a]	LAMP
**73**	HTLV-1+2	HTLV-1	HTLV-1	HTLV-1	HTLV-1	**109**	HTLV-1	**Neg**	HTLV-1	HTLV-1	**Neg**
**74**	n.d.	HTLV-1	HTLV-1	HTLV-1	HTLV-1	**110**	HTLV-1	HTLV-1	HTLV-1	HTLV-1	HTLV-1
**75**	HTLV-1	HTLV-1	HTLV-1	HTLV-1	HTLV-1	**111**	HTLV-1	HTLV-1	HTLV-1	HTLV-1	HTLV-1
**76**	HTLV-1	HTLV-1	HTLV-1	HTLV-1	HTLV-1	**112**	HTLV-1	HTLV-1	HTLV-1	HTLV-1	HTLV-1
**77**	HTLV-1	HTLV-1	HTLV-1	HTLV-1	HTLV-1	**113**	HTLV-1	**Neg**	HTLV-1	HTLV-1	**Neg**
**78**	HTLV-1	HTLV-1	HTLV-1	HTLV-1	HTLV-1	**114**	HTLV-1	HTLV-1	HTLV-1	HTLV-1	HTLV-1
**79**	HTLV-2	**Neg**	HTLV-2	HTLV-2	HTLV-2	**115**	HTLV-1	HTLV-1	HTLV-1	HTLV-1	HTLV-1
**80**	HTLV-2	HTLV-2	HTLV-2	HTLV-2	HTLV-2	**116**	HTLV-1	HTLV-1	HTLV-1	HTLV-1	HTLV-1
**81**	HTLV-2	HTLV-2	HTLV-2	HTLV-2	HTLV-2	**117**	IND	**Neg**	HTLV-1	HTLV-1	**Neg**
**82**	HTLV-2	**Neg**	HTLV-2	HTLV-2	HTLV-2	**118**	HTLV-1	**Neg**	HTLV-1	HTLV-1	HTLV-1
**83**	HTLV-2	**Neg**	HTLV-2	HTLV-2	HTLV-2	**119**	HTLV-1	**Neg**	**Neg**	HTLV-1	HTLV-1
**84**	HTLV-2	HTLV-2	HTLV-2	HTLV-2	HTLV-2	**120**	HTLV-1	HTLV-1	HTLV-1	HTLV-1	HTLV-1
**85**	HTLV-2	HTLV-2	HTLV-2	HTLV-2	HTLV-2	**121**	HTLV-1	**Neg**	**Neg**	HTLV-1	HTLV-1
**86**	HTLV-2	HTLV-2	HTLV-2	HTLV-2	HTLV-2	**122**	HTLV	**Neg**	HTLV-1	HTLV-1	HTLV-1
**87**	HTLV-2	HTLV-2	HTLV-2	HTLV-2	HTLV-2	**123**	HTLV-1	HTLV-1	HTLV-1	HTLV-1	HTLV-1
**88**	n.d.	HTLV-2	HTLV-2	HTLV-2	HTLV-2	**124**	HTLV-1	HTLV-1	HTLV-1	HTLV-1	HTLV-1
**89**	HTLV-2	**Neg**	**Neg**	HTLV-2	HTLV-2	**125**	HTLV-1	**Neg**	HTLV-1	HTLV-1	HTLV-1
**90**	n.d.	**Neg**	HTLV-2	HTLV-2	**Neg**	**126**	HTLV-1+2	HTLV-1	HTLV-1	HTLV-1	HTLV-1
**91**	HTLV-2	**Neg**	HTLV-2	HTLV-2	**Neg**	**127**	HTLV-1	**Neg**	HTLV-1	HTLV-1	HTLV-1
**92**	HTLV-2	**Neg**	**Neg**	HTLV-2	**Neg**	**128**	IND	**Neg**	HTLV-1	HTLV-1	**Neg**
**93**	HTLV-2	**Neg**	**Neg**	HTLV-2	**Neg**	**129**	HTLV-1	HTLV-1	HTLV-1	HTLV-1	HTLV-1
**94**	HTLV-2	**Neg**	**Neg**	HTLV-2	**Neg**	**130**	HTLV-1	HTLV-1	HTLV-1	HTLV-1	HTLV-1
**95**	HTLV-2	**Neg**	**Neg**	HTLV-2	HTLV-2	**131**	HTLV-1	HTLV-1	HTLV-1	HTLV-1	HTLV-1
**96**	HTLV-2	**Neg**	**Neg**	HTLV-2	**Neg**	**132**	HTLV-1	HTLV-1	HTLV-1	HTLV-1	HTLV-1
**97**	HTLV-2	**Neg**	**Neg**	HTLV-2	HTLV-2	**133**	HTLV-1	HTLV-1	HTLV-1	HTLV-1	HTLV-1
**98**	HTLV-1	**Neg**	HTLV-1	HTLV-1	**Neg**	**134**	HTLV-1	HTLV-1	HTLV-1	HTLV-1	HTLV-1
**99**	HTLV-1	HTLV-1	HTLV-1	HTLV-1	HTLV-1	**135**	HTLV-1	HTLV-1	HTLV-1	HTLV-1	HTLV-1
**100**	HTLV-1	**Neg**	HTLV-1	HTLV-1	HTLV-1	**136**	HTLV-1	HTLV-1	HTLV-1	HTLV-1	HTLV-1
**101**	HTLV-1	HTLV-1	HTLV-1	HTLV-1	HTLV-1	**137**	**HTLV-2**	HTLV-1	HTLV-1	HTLV-1	HTLV-1
**102**	HTLV-1	HTLV-1	HTLV-1	HTLV-1	HTLV-1	**138**	HTLV-1	HTLV-1	HTLV-1	HTLV-1	HTLV-1
**103**	HTLV-1	**Neg**	HTLV-1	HTLV-1	**Neg**	**139**	HTLV-1	HTLV-1	HTLV-1	HTLV-1	HTLV-1
**104**	HTLV-1	**Neg**	HTLV-1	HTLV-1	**Neg**	**140**	HTLV-1	HTLV-1	HTLV-1	HTLV-1	HTLV-1
**105**	HTLV-1	**Neg**	**Neg**	HTLV-1	HTLV-1	**141**	HTLV-1	HTLV-1	HTLV-1	HTLV-1	HTLV-1
**106**	HTLV-1	**Neg**	HTLV-1	HTLV-1	**Neg**	**142**	HTLV-1	HTLV-1	HTLV-1	HTLV-1	HTLV-1
**107**	HTLV-1	**Neg**	HTLV-1	HTLV-1	**Neg**	**143**	HTLV-1	HTLV-1	HTLV-1	HTLV-1	HTLV-1
**108**	HTLV-1	HTLV-1	HTLV-1	HTLV-1	**Neg**	**144**	HTLV	HTLV-1	HTLV-1	HTLV-1	HTLV-1

Notes. ID, identification; WB, Western blot; qPCR, real-time quantitative PCR; PCR-RFLP, PCR-restriction fragment length polymorphism; LAMP, loop-mediated isothermal amplification; IND, indeterminate; n.d., not done; Neg, negative.

[a] The definitive result was determined by a positive result in any confirmatory assay, and the HTLV subtype was defined preferentially by the result from molecular assays (qPCR and PCR-RFLP).

Table S3: Overall accuracy and sensitivity of LAMP, qPCR and PCR-RFLP assays in individuals monoinfected by HTLV-1/2.

Groups		LAMP		qPCR		PCR-RFLP	
		accuracy	sensitivity	accuracy	sensitivity	accuracy	sensitivity
**Monoinfected**	**n**	**(%)**	**(%)**	**(%)**	**(%)**	**(%)**	**(%)**
**HTLV-1**	125	**93.1**	**92.0**	**88.2**	**86.4**	**97.9**	**97.6**
		(87.6-96.7)	(85.8-96.1)	(81.8-93.0)	(79.1-91.9)	(94.0-99.6)	(93.2-99.5)
**HTLV-2**	19	**95.8**	**68.4**	**91.7**	**36.8**	**95.1**	**63.2**
		(91.2-98.5)	(43.5-87.4)	(85.9-95.6)	(6.3-61.6)	(90.3-98.0)	(38.4-83.7)

Notes. The overall accuracy and sensitivity of HTLV-1/2 LAMP, real-time quantitative PCR (qPCR), and PCR-restriction fragment length polymorphism (PCR-RFLP) assays are shown in percentage. 95% confidence intervals are presented inside parenthesis.

Table S4: Overall accuracy and sensitivity of LAMP, qPCR and PCR-RFLP assays in HIV carriers coinfected with HTLV-1/2.

Groups		LAMP		qPCR		PCR-RFLP	
		accuracy	sensitivity	accuracy	sensitivity	accuracy	sensitivity
**HIV-coinfected**	**n**	**(%)**	**(%)**	**(%)**	**(%)**	**(%)**	**(%)**
**HTLV-1**	39	**90.2**	**79.5**	**87.8**	**74.4**	**88.9**	**81.6**
		(81.7-95.7)	(63.4-90.7)	(78.7-94.0)	(57.9-87.0)	(80.0-94.8)	(65.7-92.3)
**HTLV-2**	35	**79.3**	**51.4**	**84.2**	**62.7**	**80.3**	**54.3**
		(68.9-87.4)	(34.0-68.6)	(74.4-91.3)	(44.9-78.5)	(69.9-88.3)	(36.7-71.2)

Notes. The overall accuracy and sensitivity of HTLV-1/2 LAMP, real-time quantitative PCR (qPCR), and PCR-restriction fragment length polymorphism (PCR-RFLP) assays are shown in percentage. 95% confidence intervals are presented inside parenthesis.
